# FBXW7 suppresses cell proliferation, migration, and epithelial-mesenchymal transition in endometrioid ovarian carcinoma

**DOI:** 10.7150/jca.128606

**Published:** 2026-02-11

**Authors:** Ching-Chou Tsai, Chia-Yi Hsu, Jau-Ling Suen, Hung-Pin Tu, Kun-Bow Tsai, Shun-Chen Huang, Yu-Che Ou, Eing-Mei Tsai

**Affiliations:** 1 Graduate Institute of Clinical Medicine, College of Medicine, Kaohsiung Medical University, Kaohsiung City 807, Taiwan.; 2 Department of Obstetrics and Gynecology, Kaohsiung Chang Gung Memorial Hospital and Chang Gung University College of Medicine, Kaohsiung 833, Taiwan.; 3 Graduate Institute of Medicine, College of Medicine, Kaohsiung Medical University, Kaohsiung City 807, Taiwan.; 4 Department of Obstetrics and Gynecology, Kaohsiung Medical University Hospital, Kaohsiung Medical University, Kaohsiung City 807, Taiwan.; 5 Department of Public Health and Environmental Medicine, School of Medicine, College of Medicine, Kaohsiung Medical University, Kaohsiung 807, Taiwan.; 6 Department of Pathology, Kaohsiung Medical University Hospital, Kaohsiung Medical University, Kaohsiung City, Taiwan.; 7 Department of Anatomic Pathology, Kaohsiung Chang Gung Memorial Hospital and Chang Gung University, Kaohsiung 833, Taiwan.; 8 Department of Obstetrics and Gynecology, Chia-Yi Chang Gung Memorial Hospital, Chia-Yi 613, Taiwan.

**Keywords:** epithelial ovarian carcinoma, endometrioid ovarian carcinoma, epithelial-mesenchymal transition, FBXW7

## Abstract

Epithelial ovarian carcinoma is a common gynecologic malignancy. Evidence from several studies suggests that subtypes of this cancer—specifically clear-cell ovarian carcinoma and endometrioid ovarian carcinoma (ENOC)—are associated with endometriosis. FBXW7 (F-box and WD repeat domain containing 7) is a tumor suppressor and component of an E3 ubiquitin ligase complex, which is responsible for tagging proteins for proteasomal degradation. FBXW7 is one of the most frequently dysregulated proteins of the ubiquitin-proteasome system in various human cancers. Thus, we investigated whether FBXW7 dysfunction contributes to epithelial ovarian carcinoma. We found that the level of FBXW7 was lower in endometriosis-associated ovarian carcinoma, especially ENOC. Functional assays revealed that overexpression of FBXW7 inhibited the proliferation and migration of ovarian carcinoma cells, whereas FBXW7 knockdown had the opposite effect. We also found that FBXW7 expression was associated with reduced vimentin levels, accompanied by changes in epithelial-mesenchymal transition (EMT) markers.

Overexpression of FBXW7 increased E-cadherin levels while reducing N-cadherin and vimentin levels, thereby promoting the epithelial phenotype. Conversely, FBXW7 knockdown upregulated vimentin and N-cadherin levels, facilitating EMT. Co-immunoprecipitation assays indicated that FBXW7 co-precipitates with vimentin, suggesting a possible role for FBXW7 in influencing vimentin abundance. These findings highlight FBXW7 as a potential tumor suppressor in endometriosis-associated ovarian carcinoma, with effect on cell proliferation, migration, and EMT regulation. The FBXW7-vimentin association may represent previously unrecognized pathway with therapeutic relevance in ovarian carcinoma.

## Introduction

Epithelial ovarian carcinoma is a common of gynecologic malignancy. Evidence from several studies has suggested that the subtypes of epithelial ovarian carcinoma, specifically clear-cell ovarian carcinoma (CCOC) and endometrioid ovarian carcinoma (ENOC), are associated with endometriosis 1. Other studies have revealed that women with endometriosis experience a higher incidence of epithelial ovarian carcinoma, and the risk of ovarian carcinoma is 4.2-fold higher than for healthy individuals 2. Finally, endometriosis is a common comorbidity and risk factor for development of certain histologic types of ovarian carcinoma, known as endometriosis-associated ovarian carcinoma (EAOC) 3.

FBXW7 (F-box and WD repeat domain-containing 7), also known as CDC4, is the F-box protein component of the Skp1-cullin 1-F-box (SCF)-type E3 ubiquitin ligase complex. FBXW7 is the substrate-recognition component of the SCF-E3 complex. The ubiquitin-conjugating enzyme associates with the SCF-E3 complex to transfer ubiquitin to the protein substrate bound by the F-box protein, marking the substrate for degradation by the 26S proteasome 4-6. Many studies have shown that the physiological substrate of FBXW7 is a cell-cycle regulator such as c-Myc, cyclin E or c-Jun 7-10. By regulating the cellular levels of these cyclins and oncogenic proteins, FBXW7 influences numerous cellular physiological processes, including growth, proliferation, and differentiation 11-15. These regulatory functions underscore the pivotal role of FBXW7 in maintaining cellular homeostasis and preventing tumorigenesis.

FBXW7 has also been implicated in the epithelial-mesenchymal transition (EMT) in several cancers, including lung, colon, and breast by downregulating key EMT-driving factors such as Snail, Slug, and ZEB2 16-19. In addition, *FBXW7* is a frequently mutated gene in various human malignancies of the breast, ovaries, and endometrium, with a total mutation frequency of approximately 6% 7,14,20-22. *FBXW7* mutations also correlate with poor prognosis owing to increased resistance to chemotherapy 23. Loss of FBXW7 function results in dysregulation of the protein-degradation machinery, which may lead to oncogenesis 4. Thus, FBXW7 has been classified as a tumor suppressor.

Chronic inflammation and oxidative stress in endometriosis can lead to genetic and epigenetic alterations in endometroid tissue, drives EMT and contributes to oncogenesis. Consequently, EMT is considered as a driver of malignant transformation. Indeed, endometriosis is essentially a pathological state of EMT dysfunction. Its EMT activation mechanism highly overlaps with the FBXW7-regulated EMT pathway in various cancers. This provides strong evidence for FBXW7 involvement in EAOC malignant transformation. Although FBXW7 dysfunction has been implicated in various malignancies, its role in EMT in EAOC remains unclear. EMT plays a role in both the pathogenesis of endometriosis and ovarian carcinoma 20. Therefore, targeting EMT and its associated pathways could be a strategy for managing both endometriosis and ovarian carcinoma.

Based on previous reports suggesting that FBXW7 may regulate EMT associated proteins, we hypothesized that FBXW7 acts as a negative regulator of EMT in EAOC. Specifically, we speculate that altered FBXW7 expression affects the expression or stability of key EMT associated proteins. To address this hypothesis, we examined the expression level of FBXW7 in EAOC and investigated its correlation with EMT markers.

## Material and Methods

### Patient samples

Clinical specimens were collected from female patients who met the inclusion criteria and were diagnosed between 2010 and 2019 at the Kaohsiung Chang Gung Memorial Hospital, Kaohsiung, Taiwan. (IRB No.: 201901932B0). These patients were diagnosed with CCOC, ENOC, or endometriosis. Ultimately, 20 CCOC patients, 70 ENOC patients, and 18 patients with endometriosis were recruited for this study.

Paraffin sections of endometriosis and ovarian carcinoma tissues were collected from the Tissue Bank Core Lab and Biobank of Chang Gung Medical Foundation, Kaohsiung Chang Gung Memorial Hospital. The study was approved by the Institutional Review Board of Chang Gung Memorial Hospital, and written informed consent for participation was obtained from each participant (IRB No.: 201901932B0).

### Immunohistochemistry

For immunohistochemical staining, formalin-fixed and paraffin-embedded tissue sections were deparaffinized, hydrated, and treated with 3% hydrogen peroxide in methanol to eliminate endogenous peroxidase activity. After blocking with Bovine serum albumin (BSA), specimens on the slides were hybridized with anti-FBXW7 (ab109617, 1:500; Abcam, MA, USA) for 2 h at room temperature, washed with phosphate-buffered saline, and incubated with biotinylated secondary antibody (1:500; DAKO, Glostrup, Denmark) for 30 min at room temperature. After thorough washing, color was developed with AEC substrate buffer (Bio SB, USA) followed by hematoxylin staining (Bio SB). The immunohistochemical staining images were acquired using microscopy.

### Cell lines and culture

Human ovarian carcinoma cell lines TOV21G, TOV112D, JHOC5 and OVK18 were purchased from the American Type Culture Collection (Rockville, MD, USA). The immortalized human endometriotic cell line 12Z was purchased from Applied Biological Materials (T0764; Viking Way, Richmond, BC, Canada). Normal human ovarian surface epithelial cells (IOSE) cells were a gift from Dr. Michael Chan (National Chung Cheng University, Chiayi, Taiwan). TOV21G and TOV112D cells were maintained in a 1:1 (v/v) mixture of complete Medium 199 (Gibco-Life Technologies, Grand Island, New York) and MCDB 105 medium (Sigma-Aldrich, St. Louis, MO) supplemented with 15% fetal bovine serum (FBS; Gibco-Life Technologies). JHOC5 cells were cultured in DMEM/F12 medium (1:1) supplemented with 15% FBS and non-essential amino acids at 37°C. OVK18 were cultured in MEM medium with 15% FBS at 37°C. The 12Z cells were cultured in DMEM/F12 (1:1) supplemented with 10% FBS at 37°C. IOSE cells were maintained in complete Medium 199 and MCDB 105 medium (1:1, v/v) supplemented with 10% FBS and 400 ng/ml hydrocortisone and 10 ng/ml epidermal growth factor. All cells were incubated in a humidified incubator at 37°C with a 5% CO_2_ atmosphere.

### Plasmids and transfection

The plasmids included pDONR223 and pDONR223-FBXW7-WT (a gift from Jesse Boehm, William Hahn and David Root; plasmid #81973, Addgene, Location). Cells were transfected with each plasmid using X-treme GENE HP DNA Transfection Reagent (Roche, Indianapolis, IN, USA). The small interfering RNA (siRNA) specific for FBXW7 (siFBXW7) was purchased from (GE Healthcare Dharmacon, Lafayette, CO, USA). Transfection of cells with siFBXW7 was carried out with DharmaFECT 1 transfection reagent (GE Healthcare Dharmacon).

### Cell viability analysis

The Cell Counting Kit-8 (CCK-8) assay (Honeywell Fluka; Thermo Fisher Scientific, Rockford, IL) was used to measure cell viability. Briefly, ovarian carcinoma cell lines were seeded into 96-well plates at a density of 5 × 10^3^ cells/well in a total volume of 100 μl medium with 15% (v/v) FBS, transfected with a plasmid, siRNA, or empty vector (control) and then incubated at 37°C for 24 h in a 5% CO_2_ atmosphere. The following day, cell viability was measured with the CCK-8 assay. Cells were maintained for an additional 2 h at 37°C, and the absorbance at 450 nm was read with a microplate reader (reference, 650 nm; Multiskan EX, Thermo Fisher Scientific). Three independent experiments were performed for each analysis.

### Migration assay

The migration of cells transfected with FBXW7 or siFBXW7 was assessed using transwell inserts with a pore size of 8 µm (BD Biosciences, Franklin Lakes, NJ, USA). Transited cells (1 × 10^4^) in 300 µl serum-free medium were plated into each migration chamber, whereas 700 µl of medium with 15% FBS was added to the lower well of the 24-well plate. Following incubation for 18 h, cells that had invaded into the reverse side of the transwell membrane were fixed with 4% paraformaldehyde and stained with 0.1% crystal violet at room temperature for 20 min. Cell migration was quantified by counting three random fields of view under a phase-contrast microscope. In each case, three independent experiments were performed.

### Western blotting

Protein was extracted from cells with RIPA lysis buffer (Thermo Fisher Scientific) and then quantified with the Pierce BCA Protein Assay kit (Thermo Fisher Scientific). Proteins were separated by SDS-PAGE (10% polyacrylamide gel) at 100 V and electrotransferred onto a polyvinylidene difluoride membrane at 90 V for 1.5 h. Membranes were blocked at room temperature for 10 min with blocking buffer (T-pro Technology New Taipei County, Taiwan). After washing three times with Tris-buffered saline (TBS) containing 5% (w/v) Tween 20, each membrane was incubated overnight at 4°C with a primary antibody: anti-FBXW7 (ab109617, 1:500; Abcam), anti- E-cadherin (GTX100443,1:1000; GeneTex, San Antonio, TX, USA), anti- N-cadherin (GTX127345,1:1000; GeneTex, San Antonio, TX, USA), anti-vimentin (V9, 1:1000; Santa Cruz Biotechnology, Dallas, TX, USA), or anti-actin (A2228, 1:5000; Sigma-Aldrich; Merck KGaA). Each membrane was washed three times with the TBS and incubated at room temperature for 1 h with the secondary antibody: horseradish peroxidase-conjugated goat anti-rabbit (1:5000) or anti-mouse antibody (1:5000) (Santa Cruz Biotechnology, Dallas, TX, USA). Immunodetection was achieved with enhanced chemiluminescence reagents (EMD Millipore).

### RNA extraction, cDNA synthesis and Quantitative real-time PCR

Total RNA was isolated from cells using Trizol (Thermo Fisher Scientific). RNA was reverse transcribed using the Deoxy + HiSpec RT kit (cat. FYT501-100R; Yeastern Biotech, Taipei, Taiwan). The qPCR analysis was performed using the ORA qPCR Green ROX L Mix (highQu, Kraichtal, Germany) with the Applied Biosystems 7500 Real-Time PCR System (Thermo Fisher Scientific), and amplified qPCR products were quantified and normalized using 18S rRNA as the control. The primers were synthesized by Genomics BioSci and Tech (New Taipei City, Taiwan): *18S* forward, 5'-CATGGCCGTTCTTAGTTGGT-3' and reverse, 5'-CGCTGAGCCAGTCAGTGTAG-3'; *E-cadherin* forward, 5'-GAAAGCGGCTGATACTGACC-3' and reverse, 5'-CGTACATGTCAGCCGCTTC-3'; *FBXW7* forward, 5'-CAGCAGTCACAGGCAAATGT-3' and reverse, 5'-GCATCTCGAGAACCGCTAAC-3'; *N-cadherin* forward, 5'-TGTTTGACTATGAAGGCAGTG-3' and reverse, 5'-TCAGTCATC ACCTCCACCAT-3'; *E-cadherin* forward, 5'-GAAAGCGGCTGATACTGACC-3' and reverse, 5'-CGTACATGTCAGCCGCTTC-3'; and *vimentin* forward, 5'-AGGCAAAGCAGGAGTCCACTGA-3' and reverse, 5'-ATCTGGCGTTCCAGGGACTCAT-3'. Relative expression was calculated with the 2^-ΔΔCq^ method.

### Co-immunoprecipitation assay

Epithelial ovarian carcinoma cells were transfected with a plasmid encoding FBXW7 and cultured in a 10-cm dish at 37°C for 48 h, after which the cells were collected. Cell extracts were prepared using RIPA lysis buffer and subsequently centrifuged at 15000 × *g* for 10 min at 4°C. The supernatants were used for the Co-immunoprecipitation assay. Each supernatant was incubated with a FBXW7-specific polyclonal antibody for 4 h at 4°C. Protein A/G beads were added to each supernatant, with incubation for an additional 12 h at 4°C. The immunocomplexes were collected by centrifugation at 15000 × *g* for 5 min at 4°C, and each supernatant was discarded. The immunocomplexes in the resultant pellet were washed with RIPA lysis buffer, dissolved in SDS-PAGE sample buffer, and subjected to SDS-PAGE. The proteins were detected by western blotting.

### Statistical analysis

The Spearman rank correlation was used to assess the relationship between two continuous variables. All statistical analyses were done with PASW 18 release 18.0.0 (SPSS, Chicago, IL). Data are presented as the mean ± standard deviation from at least three independent experiments with Prism 7 software (GraphPad, La Jolla, CA, USA). For the *in vitro* cell migration assays, groups were compared using the Mann-Whitney U test. For all other results, differences among multiple groups were analyzed with one-way analysis of variance followed by Tukey's honest significant difference test. Differences between two groups was analyzed with the Student's t-test. Significance was defined as *P* < 0.05 for all tests.

## Results

### FBXW7 expression is elevated in clear-cell carcinoma and endometriosis compared to endometrioid carcinoma

**Figure 1:**
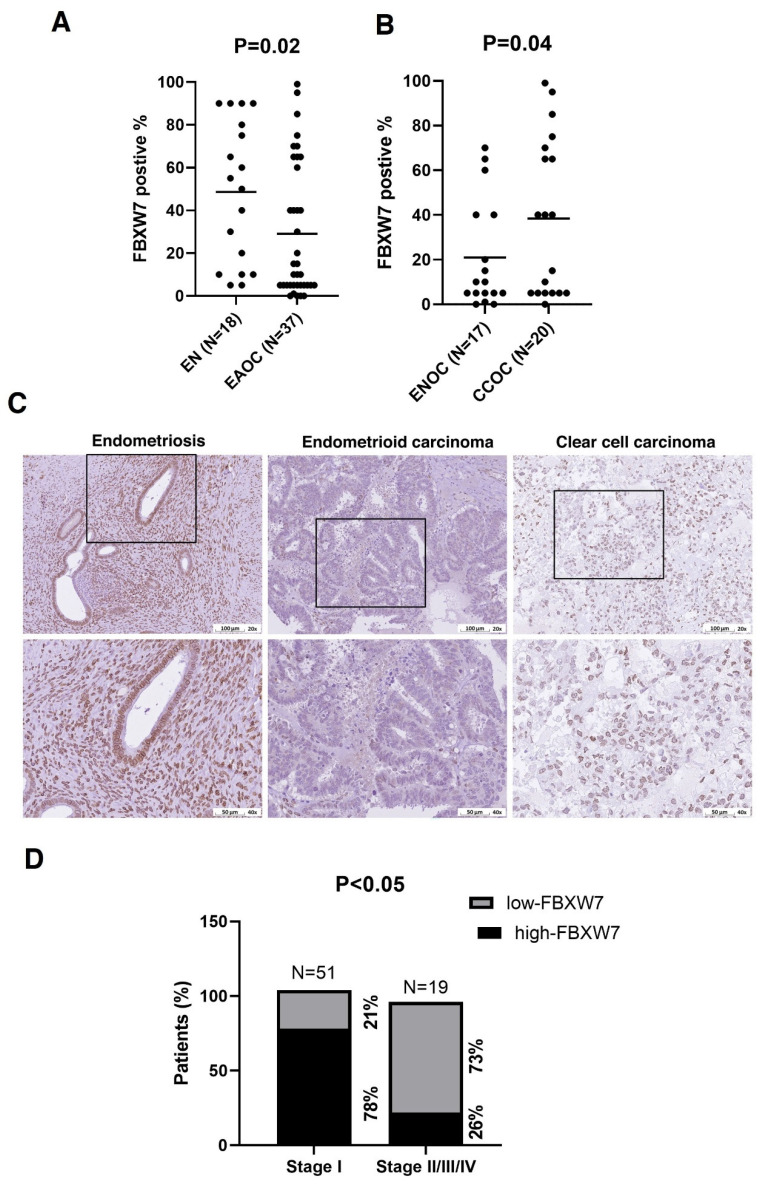
** Lower FBXW7 abundance in endometriosis-associated ovarian carcinoma tissues.** (A, B) Immunohistochemical staining for FBXW7 in endometriosis and EAOC tissues. Interrelationships were analyzed with SPSS software. (C) Representative images of FBXW7 immunostaining in ovarian cancer tissues. (D) FBXW7 abundance is substantially lower in ~73% of distal metastases.

We first investigated the clinical relevance of FBXW7 in EAOC. FBXW7 levels were lower in samples of EAOC tissue compared with endometriotic tissue (Figure 1A). We further divided EAOC samples into two types, namely CCOC and ENOC, and analyzed FBXW7 level. FBXW7 was downregulated in ENOC compared with CCOC (Figure 1B and C). We next assessed the relationship between FBXW7 level and the clinical characteristics of ENOC patients (see Table 1). FBXW7 level correlated with tumor stage (*χ*^2^ test, *P* <0.05) (Figure 1D) in ENOC. Survival analysis was performed (data not shown) but showed no significant association. These findings suggest that FBXW7 levels were associated with tumor stage, which may reflect a role in tumor progression, although additional mechanistic studies are needed to clarify its biological significance.

### Effect of FBXW7 on cell viability and migration *in vitro*

**Figure 2:**
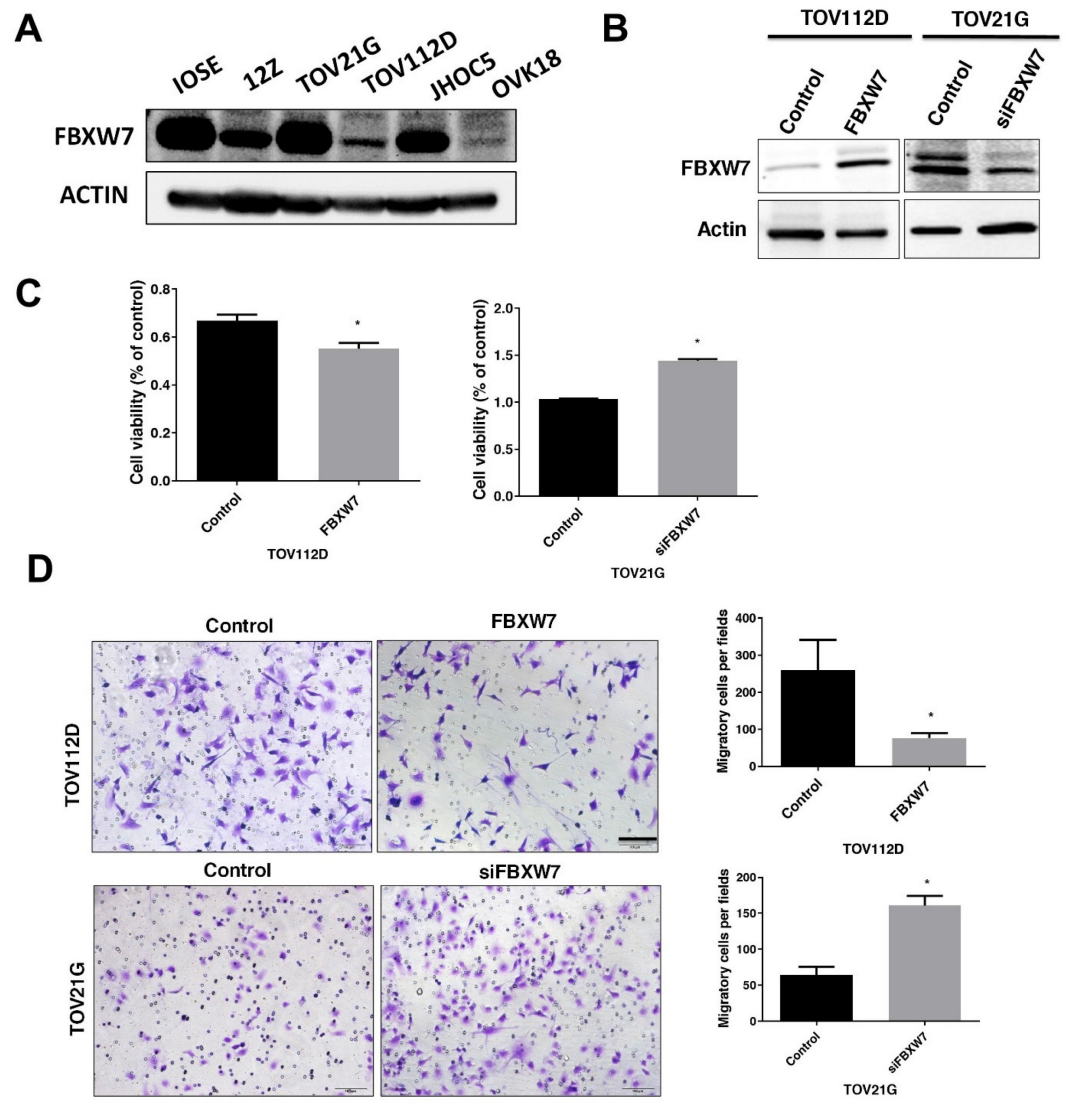
** FBXW7 levels in epithelial ovarian carcinoma cells.** (A) FBXW7 abundance was analyzed by western blotting in cell lines IOSE, 12Z, TOV21G, TOV112D, JHOC5, and OVK18. Actin served as the internal control. (B) Transfection of TOV112D cells with a *FBXW7*-expressing plasmid (pDONR223-*FBXW7*-WT) and of TOV21G cells with a plasmid encoding an *FBXW7*-specific siRNA (si*FBXW7*) and analysis of FBXW7 protein by western blotting. (C, D) FBXW7 abundance affects both cell viability (C) and migration capacity (D). *P < 0.05. Scale bars=200 μm. Data are presented as the mean ± standard deviation from three independent experiments.

To investigate the potential role of FBXW7 in EAOC. Further, we measured FBXW7 level in human normal ovarian cell lines, IOSE cells from ovarian surface epithelium, the immortalized human endometriotic cell line 12Z, and the ovarian carcinoma cell lines TOV21G, TOV112D, JHOC5 and OVK18. We measured FBXW7 level in a human normal ovarian cell line (IOSE), endometriotic cell line (12Z), and the ovarian carcinoma cell lines TOV21G, TOV112D, JHOC5 and OVK18. FBXW7 level was substantially lower in TOV112D cells compared with IOSE cells. In contrast, FBXW7 level was high in TOV21G cells (Figure 2A). Compared with IOSE cells, FBXW7 level was substantially lower in TOV112D cells, yet it was essentially equivalent to IOSE cells in TOV21G cells (Figure 2A). Consequently, based on these differences in FBXW7 levels among the various ovarian carcinoma cells, we knocked down *FBXW7* in TOV21G cells with a siFBXW7 and overexpressed *FBXW7* by introducing a *FBXW7*-containing plasmid into TOV112D cells for functional assay (Figure 2B). As shown in Figure 2C and D, cell growth and migration were attenuated by FBXW7 overexpression. In contrast, cell proliferation and migration were promoted upon downregulation of *FBXW7* mRNA. These findings suggested that FBXW7 contributes to the modulation of both the viability and migration of ovarian carcinoma cells.

### FBXW7 affects EMT progression in ovarian carcinoma cells

Based on the findings presented in Figure 1 that FBXW7 level may correlate with the clinical stages of ovarian carcinoma, and the known associated with EMT and tumor progression. We further examined whether alterations in FBXW7 levels were accompanied by changes in EMT markers.

**Figure 3:**
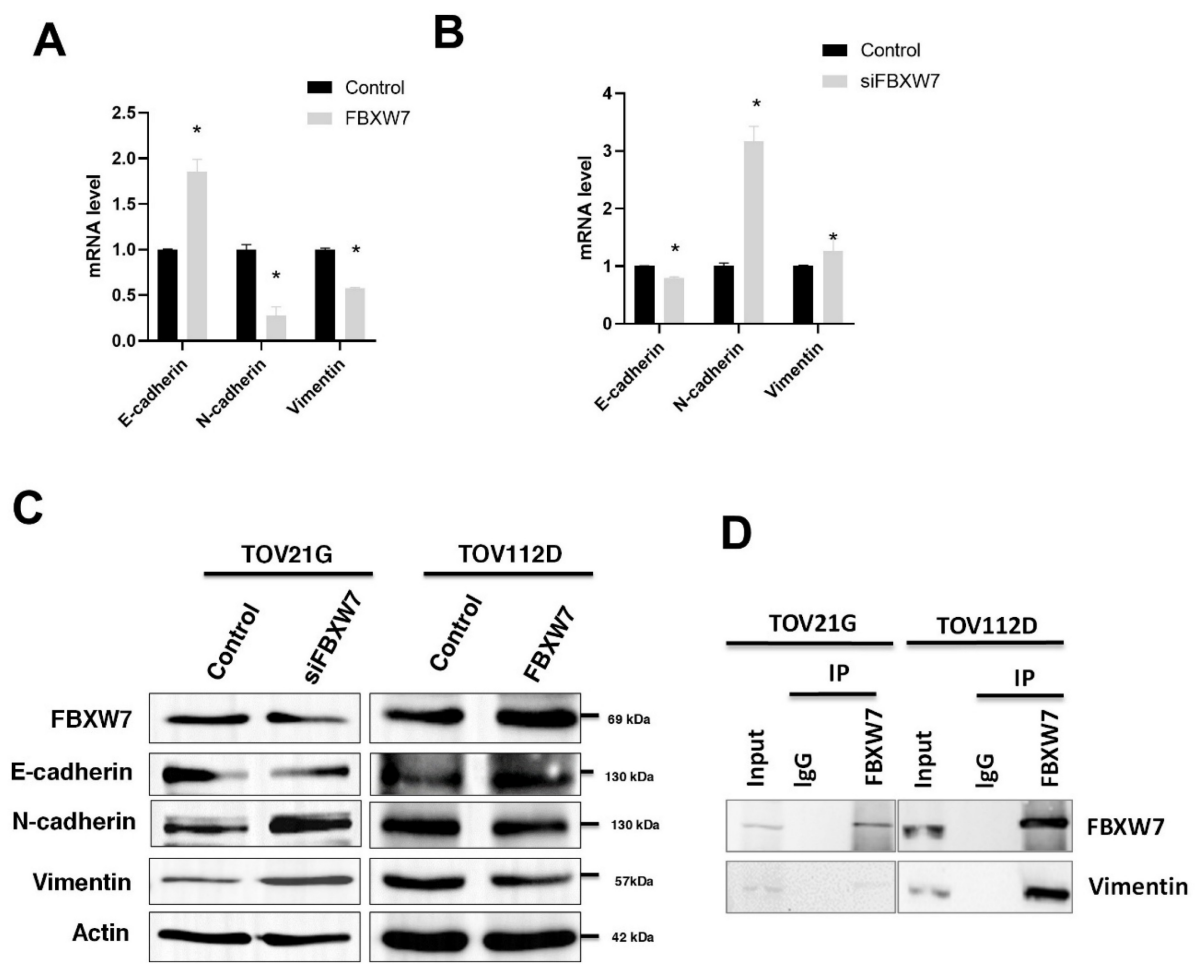
** FBXW7 interacts with vimentin.** (A, B) Quantitative real-time PCR analysis of cellular mRNA levels of *E-cadherin, N-cadherin*, and *Vimentin* in ovarian carcinoma cell line TOV112D overexpressing *FBXW7* (A) and in TOV21G cells in which *FBXW7* was knockdown with siFBXW7 (B). *P < 0.05. Data are presented as the mean ± standard deviation from three independent experiments. (C) Western blotting for FBXW7, vimentin, N-cadherin, and E-cadherin in ovarian carcinoma cells with FBXW7 overexpression or knockdown. Actin served as the internal control. (D) Co-immunoprecipitation (Co-IP) assays were performed using ovarian carcinoma cell lines. Cell lysates were immunoprecipitated with an anti-FBXW7 antibody or control IgG using Protein A/G agarose beads. The immunoprecipitated complexes were subjected to western blotting and probed with anti-vimentin and anti-FBXW7 antibodies.

To evaluate whether FBXW7 level affects EMT, we knocked down or overexpressed *FBXW7* in the ovarian carcinoma cell lines TOV112D and TOV21G. *FBXW7* overexpression in TOV112D cells led to increased expression of *E-cadherin* but decreased expression of *N-cadherin* and *vimentin* (Figure 3A). The data indicate a shift toward a more epithelial phenotype, with increased cell adhesion and potentially reduced migratory capacity.

In contrast*, FBXW7* knockdown in TOV21G cells led to decreased expression of *E-cadherin* but increased expression of *N-cadherin* and *vimentin* (Figure 3B)*.* This finding indicates a shift toward a more mesenchymal phenotype, which is associated with increased invasiveness. Western blotting was also performed to analyze the expression of E-cadherin, N-cadherin, and vimentin. FBXW7 knockdown in TOV21G cells led to decreased expression of E-cadherin but increased expression of N-cadherin and vimentin. In contrast, FBXW7 overexpression led to increased E-cadherin expression, accompanied by a decrease in N-cadherin and vimentin (Figure 3C). This supports the involvement of FBXW7 in regulating EMT markers and influencing the degree of EMT phenotype transition. The results suggested that FBXW7 plays a role in regulating the expression of markers associated with EMT and indicated that FBXW7 may play a role in regulating the cellular level of vimentin, a protein associated with the mesenchymal phenotype (Figure 3C).

### FBXW7 is associated with vimentin in the same protein complex

FBXW7 is the substrate-recognition component of the SCF-E3 ubiquitin ligase complex. We performed co-immunoprecipitation (Co-IP) assays to investigate whether FBXW7 is associated with vimentin regulation in ovarian carcinoma cells. The results showed that FBXW7 co-precipitates with vimentin, suggesting they are present in a common protein complex and a potential role for FBXW7 in regulating vimentin. However, additional functional and biochemical experiments would be required to determine whether FBXW7 directly affects vimentin stability (Figure 3D).

## Discussion

Emerging evidence indicates that ENOC and CCOC are frequently associated with endometriosis. EAOC, particularly clear cell and endometrioid subtypes, is widely believed to arise from malignant transformation of endometriotic lesions. Our results suggest that FBXW7 is downregulated in EAOC, especially ENOC. Furthermore, the loss of FBXW7 correlated with advanced stages of ENOC, suggesting that loss of FBXW7 function may drive malignant progression (Fig 1). We further demonstrated that FBXW7 overexpression significantly inhibited the proliferation and migration capacity of ovarian carcinoma cells, whereas FBXW7 knockdown promoted their migration capacity.

Dysfunction of EMT in epithelial cells of the uterus and ovary has been implicated in the onset of various gynecological disorders, including adenomyosis, endometriosis, and the initiation and spread of reproductive-tract malignancies 24. For endometriotic cells or tumor cells to migrate and form metastases, the cells must undergo changes in cell-cell adhesion, remodel the cell matrix at adhesion sites, and migrate through the extracellular matrix in response to a chemoattractive signal—a process that is characteristic of EMT 25. FBXW7, a member of the F-box protein family, functions as a tumor suppressor in various human cancers by targeting the ubiquitin-mediated degradation of several proteins, including Snail, c-Myc, and Notch 1. Many of these targeted proteins are oncoproteins, i.e., proteins that, when dysregulated, promote cell growth, division and EMT. FBXW7 has been implicated in various malignancies, yet its role in EMT in ENOC remains unclear.

In our study, knockdown of FBXW7 was associated with increased levels of vimentin and N-cadherin, which are canonical mesenchymal markers commonly linked to EMT-related phenotypic changes. In contrast, the epithelial marker E-cadherin was downregulated in FBXW7-knockdown ovarian carcinoma cells. Furthermore, co-immunoprecipitation data revealed that FBXW7 and vimentin are present within the same protein complex, suggesting that FBXW7 is functionally linked to vimentin. However, this result indicated an association that FBXW7 and vimentin are associated within a protein complex rather than direct evidence of substrate regulation. Therefore, the relationship between FBXW7 and vimentin should be interpreted as a protein complex-level association, and its underlying molecular mechanism in EMT regulation remains to be further clarified.

In the study, we identify FBXW7 as a potential tumor suppressor in EAOC, whose downregulation is associated with EMT activation and cancer carcinogenesis. The findings suggest that FBXW7 is critical in regulating cancer-cell proliferation, migration, and EMT. It is association with vimentin suggests the existence of a regulatory relationship that requires further verification. Our future studies will focus on elucidating the mechanistic basis of the FBXW7-vimentin relationship and determining whether vimentin is a direct substrate of FBXW7, as well as exploring the potential therapeutic relevance of restoring FBXW7 function in ovarian carcinoma.

## Figures and Tables

**Table 1:** Demographic information for the three patient groups.

	Group		
Variable	Endometriosis	EAOC	
		CCOC	ENOC
**N**	18	20	70
**Age, years** (mean±SD)	39.39±9.15	46.56±6.57	47.65±11.81
**FIGO Stage**			
I		15 (%)	44 (75%)
II		2 (%)	11 (10%)
III		3 ()	11 (15%)
IV		0 (0)	5
**Grade**			
1		0	32 (17.1%)
2		7	31 (%)
3		13	9 (%)

SD, standard deviation; FIGO, Federation International of Gynecology and Obstetrics; EAOC: endometriosis-associated ovarian carcinoma; CCOC: clear-cell ovarian carcinoma; ENOC: endometrioid ovarian carcinoma

## Data Availability

The authors confirm that the data supporting the findings of this study are available within the article.

## References

[1] Pearce CL, Templeman C, Rossing MA, Lee A, Near AM, Webb PM, Nagle CM, Doherty JA, Cushing-Haugen KL, Wicklund KG (2012). Association between endometriosis and risk of histological subtypes of ovarian cancer: a pooled analysis of case-control studies. The lancet oncology.

[2] Heidemann LN, Hartwell D, Heidemann CH, Jochumsen KM (2014). The relation between endometriosis and ovarian cancer-a review. Acta obstetricia et gynecologica Scandinavica.

[3] Mandai M, Suzuki A, Matsumura N, Baba T, Yamaguchi K, Hamanishi J, Yoshioka Y, Kosaka K, Konishi I (2012). Clinical management of ovarian endometriotic cyst (chocolate cyst): diagnosis, medical treatment, and minimally invasive surgery. Current Obstetrics and Gynecology Reports.

[4] Fujii Y, Yada M, Nishiyama M, Kamura T, Takahashi H, Tsunematsu R, Susaki E, Nakagawa T, Matsumoto A, Nakayama KI (2006). Fbxw7 contributes to tumor suppression by targeting multiple proteins for ubiquitin-dependent degradation. Cancer science.

[5] Kipreos ET, Pagano M (2000). The F-box protein family. Genome biology.

[6] Takeishi S, Nakayama KI (2014). Role of Fbxw7 in the maintenance of normal stem cells and cancer-initiating cells. British journal of cancer.

[7] Strohmaier H, Spruck CH, Kaiser P, Won K-A, Sangfelt O, Reed SI (2001). Human F-box protein hCdc4 targets cyclin E for proteolysis and is mutated in a breast cancer cell line. Nature.

[8] Popov N, Schülein C, Jaenicke LA, Eilers M (2010). Ubiquitylation of the amino terminus of Myc by SCFβ-TrCP antagonizes SCFFbw7-mediated turnover. Nature cell biology.

[9] Nateri AS, Riera-Sans Ls, Costa CD, Behrens A (2004). The ubiquitin ligase SCFFbw7 antagonizes apoptotic JNK signaling. Science.

[10] Welcker M, Clurman BE (2008). FBW7 ubiquitin ligase: a tumour suppressor at the crossroads of cell division, growth and differentiation. Nature Reviews Cancer.

[11] Welcker M, Orian A, Grim JA, Eisenman RN, Clurman BE (2004). A nucleolar isoform of the Fbw7 ubiquitin ligase regulates c-Myc and cell size. Current Biology.

[12] Welcker M, Orian A, Jin J, Grim JA, Harper JW, Eisenman RN, Clurman BE (2004). The Fbw7 tumor suppressor regulates glycogen synthase kinase 3 phosphorylation-dependent c-Myc protein degradation. Proceedings of the National Academy of Sciences.

[13] Yada M, Hatakeyama S, Kamura T, Nishiyama M, Tsunematsu R, Imaki H, Ishida N, Okumura F, Nakayama K, Nakayama KI (2004). Phosphorylation-dependent degradation of c-Myc is mediated by the F-box protein Fbw7. The EMBO journal.

[14] Rajagopalan H, Jallepalli PV, Rago C, Velculescu VE, Kinzler KW, Vogelstein B, Lengauer C (2004). Inactivation of hCDC4 can cause chromosomal instability. Nature.

[15] Hoeck JD, Jandke A, Blake SM, Nye E, Spencer-Dene B, Brandner S, Behrens A (2010). Fbw7 controls neural stem cell differentiation and progenitor apoptosis via Notch and c-Jun. Nature neuroscience.

[16] Hu Z, Wu Y, Sun X, Tong Y, Qiu H, Zhuo E (2024). ARMCX1 inhibits lung adenocarcinoma progression by recruiting FBXW7 for c-Myc degradation. Biology Direct.

[17] Xiao G, Li Y, Wang M, Li X, Qin S, Sun X, Liang R, Zhang B, Du N, Xu C (2018). FBXW 7 suppresses epithelial-mesenchymal transition and chemo-resistance of non-small-cell lung cancer cells by targeting snai1 for ubiquitin-dependent degradation. Cell proliferation.

[18] Li N, Babaei-Jadidi R, Lorenzi F, Spencer-Dene B, Clarke P, Domingo E, Tulchinsky E, Vries RG, Kerr D, Pan Y (2019). An FBXW7-ZEB2 axis links EMT and tumour microenvironment to promote colorectal cancer stem cells and chemoresistance. Oncogenesis.

[19] Zhang Y, Zhang X, Ye M, Jing P, Xiong J, Han Z, Kong J, Li M, Lai X, Chang N (2018). FBW7 loss promotes epithelial-to-mesenchymal transition in non-small cell lung cancer through the stabilization of Snail protein. Cancer letters.

[20] Akhoondi S, Sun D, von der Lehr N, Apostolidou S, Klotz K, Maljukova A, Cepeda D, Fiegl H, Dofou D, Marth C (2007). FBXW7/hCDC4 is a general tumor suppressor in human cancer. Cancer research.

[21] Kwak EL, Moberg KH, Wahrer DC, Quinn JE, Gilmore PM, Graham CA, Hariharan IK, Harkin DP, Haber DA, Bell DW (2005). Infrequent mutations of Archipelago (hAGO, hCDC4, Fbw7) in primary ovarian cancer. Gynecologic oncology.

[22] Spruck CH, Strohmaier H, Sangfelt O, Muller HM, Hubalek M, Muller-Holzner E, Marth C, Widschwendter M, Reed SI (2002). hCDC4 gene mutations in endometrial cancer. Cancer research.

[23] Gong J, Zhou Y, Liu D, Huo J (2018). F-box proteins involved in cancer-associated drug resistance. Oncology letters.

[24] Lamouille S, Xu J, Derynck R (2014). Molecular mechanisms of epithelial-mesenchymal transition. Nature reviews Molecular cell biology.

[25] Yilmaz M, Christofori G (2009). EMT, the cytoskeleton, and cancer cell invasion. Cancer and metastasis reviews.

